# Cardiorespiratory Fitness as a Moderator of Sleep-Related Associations with Hippocampal Volume and Cognition

**DOI:** 10.3390/brainsci12101360

**Published:** 2022-10-07

**Authors:** Alfonso J. Alfini, Junyeon Won, Lauren R. Weiss, Casandra C. Nyhuis, Vadim Zipunnikov, Adam P. Spira, Teresa Liu-Ambrose, Alexander J. Shackman, J. Carson Smith

**Affiliations:** 1 National Center on Sleep Disorders Research, Division of Lung Diseases, National Heart, Lung, and Blood Institute, NIH, Bethesda, MD 20817, USA; 2 Department of Kinesiology, University of Maryland School of Public Health, College Park, MD 20742, USA; 3 Neuroscience and Cognitive Science Program, University of Maryland, College Park, MD 20742, USA; 4 Department of Public Health Sciences, Pennsylvania State University College of Medicine, Hershey, PA 17033, USA; 5 Department of Biostatistics, Johns Hopkins Bloomberg School of Public Health, Baltimore, MD 21205, USA; 6 Department of Mental Health, Johns Hopkins Bloomberg School of Public Health, Baltimore, MD 21205, USA; 7 Center on Aging and Health, Johns Hopkins Bloomberg School of Public Health, Baltimore, MD 21205, USA; 8 Department of Psychiatry and Behavioral Sciences, Johns Hopkins School of Medicine, Baltimore, MD 21205, USA; 9 Department of Physical Therapy, University of British Columbia, Vancouver, BC V6T 1Z3, Canada; 10 Djavad Mowafaghian Centre for Brain Health, University of British Columbia, Vancouver, BC V6T 1Z3, Canada; 11 Centre for Hip Health and Mobility, Vancouver Coastal Health Research Institute, Vancouver, BC V5Z 1M9, Canada; 12 Department of Psychology, University of Maryland, College Park, MD 20742, USA; 13 Maryland Neuroimaging Center, University of Maryland, College Park, MD 20742, USA

**Keywords:** sleep, actigraphy, brain volume, heart rate recovery, aging

## Abstract

The objective of this study was to understand the associations of sleep and cardiorespiratory fitness with hippocampal volume and global cognition among older adults (*n* = 30, age = 65.8 years, female = 73.3%). Wrist actigraphy provided objective measures of nighttime sleep including sleep duration, average wake bout length (WBL; sleep disturbance), and wake-to-sleep transition probability (WTSP; sleep consolidation). Cardiorespiratory fitness was quantified via cycle exercise using a modified heart rate recovery approach. Magnetic resonance imaging was used to determine hippocampal volume and the Mini-Mental State Examination was used to assess global cognition. Fitness moderated associations of sleep with hippocampal volume and cognitive performance, whereby the association of WBL—an index of poor sleep—with hippocampal atrophy was stronger among less-fit individuals, and the association of sleep duration with cognitive performance was stronger among more-fit individuals. Across the fitness levels, a longer WBL was associated with lower cognitive performance, and a higher WTSP—an index of more consolidated sleep—was associated with greater hippocampal volume. Sleep and fitness were unrelated to the volume of an amygdala control region, suggesting a degree of neuroanatomical specificity. In conclusion, higher cardiorespiratory fitness may attenuate sleep disturbance-related hippocampal atrophy and magnify the cognitive benefits of good sleep. Prospective studies are needed to confirm these findings.

## 1. Introduction

A greater life expectancy and lower birth rate have accelerated the pace of population aging. Recent estimates project that adults over age 65 will account for approximately 20% of the world’s population by 2050 [1]. Although increased longevity is a human success story, older age is associated with increased morbidity and diminished capacity for work and self-care [1]. Neurocognitive changes—including age-related declines in cognitive performance and brain health—reduce the opportunity for a long, productive, meaningful life and increase the risk of dementia. 

Converging lines of evidence indicate that sleep plays a pivotal role in neurocognitive aging [2,3]. Studies have shown that poor sleep quality is linked with cognitive deficits [4,5] and that both self-reported poor sleep quality and abnormal sleep duration, as well as objective indices of sleep fragmentation, are associated with cortical and subcortical brain atrophy [6,7,8]. 

Similar to poor sleep, lower cardiorespiratory fitness is implicated in poor neurocognitive outcomes in later life [9,10,11], and diminished aerobic capacity is a typical feature of the aging process, particularly among non-exercisers [12,13]. In general, older adults with higher cardiorespiratory fitness perform better on tasks of cognitive, perceptual, and motor ability than their less-fit counterparts [14,15,16]. Older master athletes—an elite group of older, highly fit, physically active adults—have better cognitive performance, greater cerebral blood flow, and larger brain volume than age-matched controls [17,18,19]. 

Although there is ample evidence that sleep and fitness enhance neurocognitive health, their potential interaction has rarely been examined. Nevertheless, an emerging body of evidence hints at potential synergistic effects. Acute exercise has been shown to enhance executive function to a greater degree among individuals with longer vs. shorter sleep duration [20], and higher levels of cardiorespiratory fitness weaken the association between self-reported sleep disturbance and cerebrospinal fluid (CSF) markers of neurodegeneration in older adults at risk of developing Alzheimer’s disease (AD) [21]. Despite this progress, the interactive consequences of sleep and fitness for brain structure and cognition in older adults remain largely unknown. Gaining an understanding of these interactions may have important implications regarding the need to make available low-risk, scalable, and cost-effective sleep and/or fitness interventions aimed at preserving neurocognitive function in older adults. 

Converging lines of observational and mechanistic evidence suggest that the hippocampus plays a crucial role in mediating the impact of physical health on memory and cognitive function, and several reports have linked hippocampal structure to insomnia and cognitive decline [22,23]. Older individuals who engage in regular fitness training exhibit larger hippocampal volume, better spatial memory, and higher levels of brain-derived neurotrophic factor (BDNF) [24,25,26]. Randomized controlled trials (RCTs) demonstrate that both exercise training and higher fitness increase hippocampal volume and boost executive function and memory performance [24,27,28,29,30]. Likewise, short-term (10 days) exercise cessation has been shown to reduce hippocampal blood flow in master athletes [31]. Taken together, these observations motivate the hypothesis that higher levels of cardiorespiratory fitness help to preserve hippocampal volume and global cognition in the face of poor sleep.

Here, we assessed the joint consequences of sleep and cardiorespiratory fitness on hippocampal volume and global cognitive function in older adults. We used wrist actigraphy to obtain objective measures of nighttime sleep in the natural environment including total sleep time (TST), mean wake bout length (WBL; an index of poor sleep), and wake-to-sleep transition probability (WSTP; an index of sleep consolidation). In the laboratory, we quantified individual differences in cardiorespiratory fitness (i.e., Modified Heart Rate Recovery, HRR_M_) and hippocampal volume. Global cognitive function was assessed using the Mini-Mental State Examination (MMSE) [32]. This novel combination of approaches allowed us to rigorously test whether higher levels of cardiorespiratory fitness attenuate sleep-related (a) hippocampal atrophy and (b) cognitive decline. To assess specificity, a parallel series of control analyses were performed for the amygdala, a neighboring region of the medial temporal lobe. We hypothesized that higher levels of cardiorespiratory fitness would mitigate the adverse associations of poor sleep (i.e., shorter sleep duration, less consolidated sleep, and longer wake bout length) with hippocampal volume and global cognition and that these effects would not generalize to the amygdala. 

## 2. Materials and Methods

### 2.1. Experimental Design and Setting

The current cross-sectional analyses were conducted in the broader context of a randomized cross-over design study, in which participants engaged in a 30 min aerobic exercise session or a sedentary control condition followed immediately by a structural MRI assessment. Participants performed each arm of the trial (exercise/control) on separate days (mean inter-session interval = 12.3 ± 17.6 days) and the order of both was randomized and counterbalanced across participants. Actigraphic sleep assessments occurred prior to the first imaging visit. To maximize reliability, analyses of the hippocampal structure employed MRI data from both visits (see below). Although the cross-over design was integral to the broader study, it is not central to the goals, implementation, or interpretation of the cross-sectional analyses that are the focus of the present report. 

### 2.2. Participants

**Figure 1:**
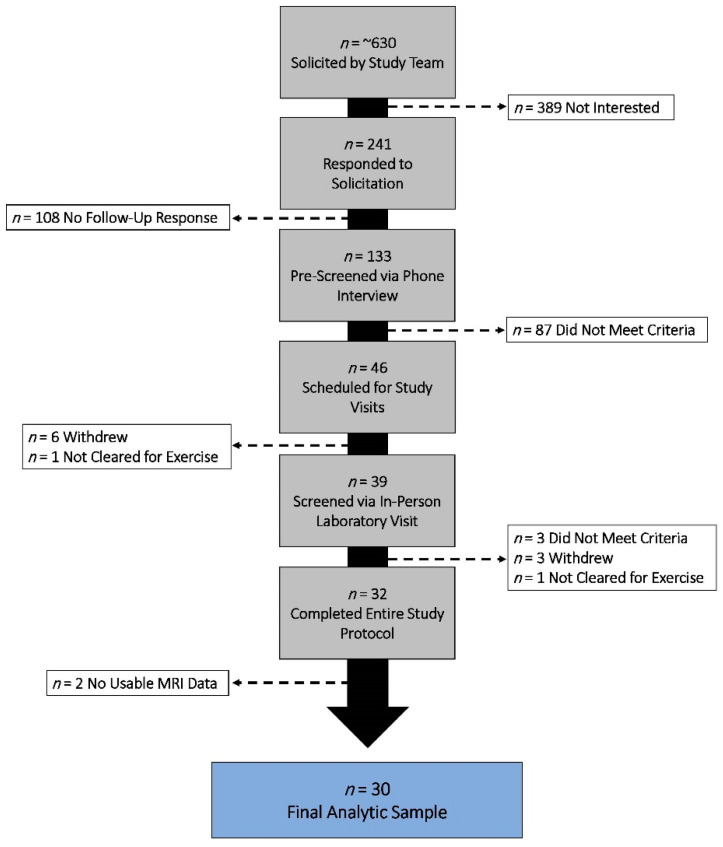
Participant recruitment, screening, exclusion, and enrollment between 2016 and 2018.

Details of the study procedures have been reported elsewhere [20,33]. Briefly, we studied 30 physically active, cognitively normal older adults (MMSE score ≥ 26, 73.3% women, age 55 to 81 years) (Table 1). Participant recruitment included in-person solicitation at local senior recreation centers, newspaper advertisements, and study fliers posted on university listservs. Individuals were screened using a structured telephone interview to ascertain health history and identify potential contraindications. Eligible individuals obtained written physician approval to engage in moderate-intensity exercise and participate in an in-person laboratory screening session, during which they were familiarized with the study procedures and provided written informed consent (Figure 1). This study was approved by the Institutional Review Board of the University of Maryland in accordance with the Helsinki Declaration.

### 2.3. Eligibility Criteria

Individuals were excluded if they reported a history of heart attack, stroke, transient ischemic attack, seizures, epilepsy, brain tumor, closed head injury, alcohol/substance abuse, psychosis; current visual/auditory limitations; or a current diagnosis of AD, atrial fibrillation, cardiovascular disease, pulmonary disease, diabetes, hypertension, Parkinson’s disease, sleep apnea, depression, anxiety, cognitive impairment, left-handedness, severe obesity (body mass index ≥ 40 kg/m^2^), self-reported low physical activity (defined here as not achieving at least 30 min of physical activity at least 3 times/week during the previous 6 months), limited English language proficiency, and MRI contraindications. 

### 2.4. Sleep Assessment

A wrist-worn actigraph (Motionlogger Watch; Ambulatory Monitoring, Inc., Ardsley, NY, USA) was used to measure sleep behavior for approximately one week. Wrist actigraphy is a valid and reliable tool for the objective measurement of sleep [34]. During the in-person screening visit, participants were trained to properly use the actigraph. On average, the participants wore the actigraph on their non-dominant wrist for 7.9 ± 3.4 days, throughout which the actigraphic data were continuously collected in one-minute epochs. Participants were trained to indicate bedtime (i.e., intention to sleep) and awakening (i.e., intention to start the day) by pressing an event-marker button on the actigraph at those times. Button-presses were then used to establish the in-bed interval. A daily sleep diary provided an ancillary source of information about the in-bed interval and actigraph removal (e.g., due to bathing) [33]. Actigraphic data and diary information were visually inspected by two trained raters to set the in-bed interval and identify invalid data (i.e., non-wear periods and periods of device malfunction). The raters were naïve to the participants’ level of cardiorespiratory fitness, and inter-rater discrepancies were resolved via consensus. Action-W software (Ambulatory Monitoring, Inc.) was used to derive the following sleep parameters: total sleep time (TST; minutes) and average wake bout length (WBL; minutes). We then calculated the wake-to-sleep transition probability (WSTP) as a measure of sleep consolidation. WSTP was defined as the probability of transitioning from an awake to a sleep state, with higher values indicating a greater sleep probability [35]. WSTP was calculated as the reciprocal of the average WBL. Each of these parameters was averaged across nights. 

### 2.5. Cognitive Assessment

The Mini-Mental State Exam (MMSE), which is a valid and reliable assessment tool, was used to measure global cognitive performance [32]. The MMSE is a well-established 11-item (yes/no) measure (score range 0–30) of global cognitive function including the domains of executive function, memory, language, and visuospatial skills [32].

### 2.6. Acute Exercise 

A single, 30 min session of moderate-intensity aerobic exercise was performed on a mechanically braked cycle ergometer (Monark 828 E, Monark Exercise AB, Vansbro Sweden). The exercise session followed a well-established cycling protocol [20] and consisted of 20 min of steady-state exercise bound between a 5 min self-directed warm-up period and cool-down period. To establish the standardized exercise intensity during the exercise session, participants maintained an RPE of 15 (associated with the verbal anchor ‘Hard’) and a pedal cadence of 60–80 revolutions per minute. Participants manually adjusted the ergometer’s resistance level to maintain the subjective effort of ‘Hard’ throughout the session while maintaining pedal cadence. The RPE was measured every five minutes and was indexed using the Borg’s 6–20 RPE scale [36]. Importantly, the RPE during the exercise session was used as the primary determinant of exercise intensity because of the evidence for its safety and validity among older adults [37]. This approach has been shown to control the relative exercise intensity among individuals who may vary in their absolute maximal capacity to perform cycle ergometer work [38]. Participants also wore a heart rate (HR) monitor (Polar RS800CX, Polar Electro, Oy, Kempele, Finland). The age-predicted max HR was calculated using the Tanaka method (208–0.7 × age) for older adults [39], which was then used to compute exercise intensity as a percentage of HR reserve (used for comparison purposes only, see Table 1). Water was provided *ad libitum* during exercise. 

### 2.7. Modified Heart Rate Recovery (HRR_M_)

Individual differences in cardiorespiratory fitness were quantified using a modified heart rate recovery (HRR_M_) approach, defined as the difference between pre- and post-exercise HR. Although this approach is not equivalent to other HRR methodologies [40,41], it is consistent with established literature, which demonstrates that (a) fitness-related improvements in vagal tone facilitate more rapid decreases in post-exercise HR towards baseline levels [42,43,44,45,46]; (b) HR remains higher (5 min) post-exercise vs. pre-exercise [47]; and (c) more-fit individuals demonstrate more rapid decreases in post-exercise HR (vs. HR at the onset of submaximal work) [43]. The HRR_M_ score was computed using the equation below, where $HR_{pre}$ as measured while participants were seated immediately before initiating exercise, and $HR_{post}$ as measured in the same position 5 min after the completion of exercise.

$$HRR_{M}=HR_{post}-HR_{pre}$$

Similar to previous studies of this nature [8,48,49], our index of cardiorespiratory fitness ($HRR_{M}$) was dichotomized using a median split (≥14 beats per minute; bpm) and analyzed as a binary variable (high fitness = 0, low fitness = 1). 

### 2.8. MRI Assessment

MRI data were acquired using a Siemens Trio Tim 3.0 Tesla MRI scanner (Erlangen, Germany) and a 32-channel head coil. Sagittal T1-weighted anatomical images were acquired using a magnetization prepared rapid gradient echo (MPRAGE) imaging sequence (TR = 1900 ms, TE = 2.3 ms, TI = 900 ms, flip angle = 9°, slice thickness = 0.9 mm, in-plane resolution = 0.9 × 0.9 mm, matrix = 256 × 256, field of view = 230 × 230).

### 2.9. MRI Data Processing

**Figure 2:**
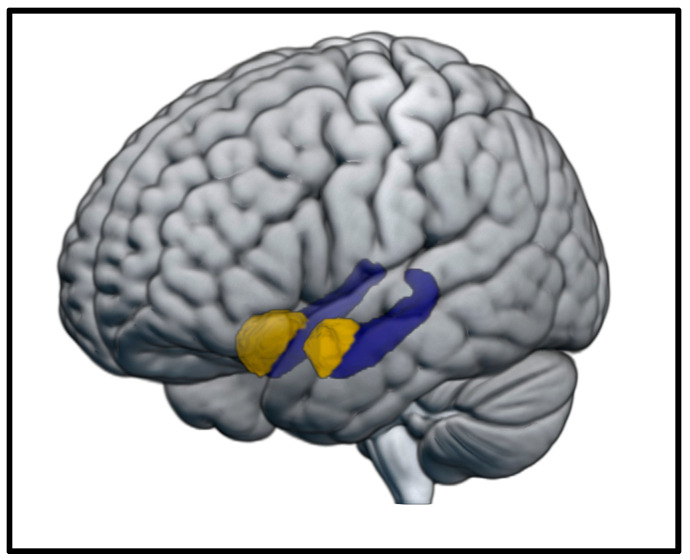
Volumetric Regions of Interest (ROIs). Bilateral hippocampus (purple) and bilateral amygdala control region (gold). Brain image and ROI visualization were generated using MRIcroGL [51].

MRI data were manually inspected before and after processing for quality assurance. T1-weighted images were processed using the FreeSurfer (version 5.3.0) automated volumetric reconstruction pipeline (recon-all), which includes tissue segmentation and intensity-based surface deformation [50]. For each participant, MRI data from the two imaging assessments were integrated using the FreeSurfer longitudinal function and normalized to stereotaxic standard space (MNI305) via nonlinear transformation. Analyses focused on bilateral hippocampal volume (Figure 2). To assess specificity, a parallel series of control analyses were performed for the amygdala (Figure 2). Regional volumetric estimates were summed across hemispheres and normalized by intracranial volume (ICV). 

### 2.10. Analytic Strategy

Analyses were conducted using Stata software (v. 15.1; StataCorp, College Station, TX, USA). Demographic and biometric characteristics for the high- and low-fitness (HRR_M_) groups were assessed using independent samples t-tests or nonparametric equality-of-means tests for continuous variables and Chi-square or Fisher exact tests for categorical variables. 

As a precursor to hypothesis testing, exploratory analyses were conducted to examine the distribution within each variable and identify potential outliers. Prior to model fitting, other statistical assumptions of linear regression were evaluated using standard techniques [52]. Ordinary least squares (OLS) linear regression was used to evaluate relations between the actigraphic sleep parameters and regional brain volume; robust regression (i.e., weighted and reweighted OLS) was used to test the associations with global cognitive performance and to confirm the results when outlier influence was suspected (i.e., values > 2.5 standard deviations from the mean). Separate models were estimated for each combination of sleep parameters and (a) regional volume or (b) global cognition (MMSE). All models controlled for potential nuisance variance in age and employed *z*-transformed continuous variables. Missing baseline HR data for one participant were imputed using the sample mean. This approach enabled us to rigorously assess the potential interactive effects of sleep and cardiorespiratory fitness (HRR_M_) on bilateral hippocampal volume. In cases where the interaction was not significant, the interaction term was removed and the analysis was repeated without HRR_M_ in the model. Parallel analyses were performed for the amygdala control region. The same approach enabled us to test the potential interactive consequences of sleep and fitness (HRR_M_) on global cognitive function (MMSE). To control for alpha inflation, the Benjamini, Krieger, and Yekutieli adaptive step-up false-discovery rate (FDR) method was used to compute the sharpened q-values for both families of analyses (i.e., (a) sleep and hippocampal volume, (b) sleep and global cognition [53]). Analyses involving the amygdala control region were not corrected for multiple comparisons. We did not adjust for or specifically evaluate the effects of biological sex for several reasons: (1) the raw volume-to-ICV fraction accounted for variance in brain volume by sex [20]; (2) we wanted to minimize the total number of statistical comparisons; (3) there was an equal number of men and women in both fitness groups; and (4) we did not hypothesize sex differences. 

## 3. Results

### 3.1. Participant Characteristics

By design, individuals with high cardiorespiratory fitness performed exercise at a lower intensity as a percentage of HR_Reserve_ (67.1 ± 18.2%) compared to individuals with low fitness (80.4 ± 21.6%) (*x*^2^(1) = 4.80, *p* = 0.028) and had a lower resting HR following the acute bout of exercise (84.9 ± 10.7 beats per minute; bpm) compared to those with low fitness (100.7 ± 11.3 bpm) (*t*(28) = −3.91, *p* = 0.001). Individuals with high fitness also slept less at night (6.2 ± 1.1 h) compared to individuals with low fitness (7.3 ± 1.0 h) (TST: *t*(28) = −2.85, *p* = 0.008). The high- and low-fitness groups did not significantly differ in any of the other measured variables (all *p*-values (*p*s) ≥ 0.062) (Table 1). 

### 3.2. Cardiorespiratory Fitness Moderated the Negative Association between Longer Wake Bout Length and Hippocampal Volume

**Figure 3:**
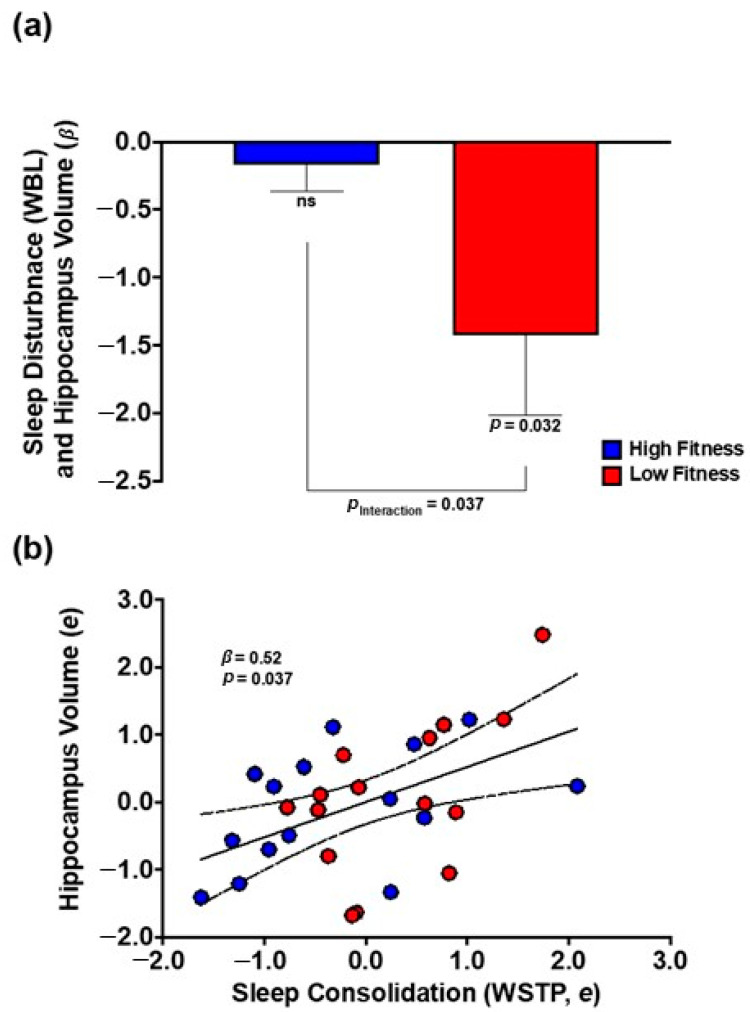
**Relations between sleep, fitness, and hippocampal volume in older adults.** (**a**) Cardiorespiratory fitness (HRR_M_) moderates the adverse association between wake bout length (WBL) and hippocampal volume. Bars depict standardized regression coefficients (β) for the sleep × fitness interaction (controlling for mean-centered age) for more-fit (*blue*) and less-fit (*red*) individuals. Among less-fit individuals (*red*), longer WBL is associated with smaller hippocampal volume. This association was not significant for more-fit individuals (*blue*). Error bars depict standard error. (**b**) Across fitness groups, greater sleep consolidation (WSTP) is associated with increased hippocampal volume (controlling for mean-centered age). *X* and *Y* axes reflect the standardized residual values for sleep consolidation (WSTP) and hippocampal volume, respectively. Dotted lines indicate the 95% confidence interval.

As expected, variations in cardiorespiratory fitness (HRR_M_) significantly moderated relations between wake bout length (WBL) and hippocampal volume (β = −1.36, 95% confidence interval [CI] = −2.45, −0.28, FDR-corrected *p* = 0.037). As shown in Figure 3a, stratified analyses demonstrated that a greater WBL was associated with smaller hippocampal volume among less-fit individuals (β = −1.42, 95% CI = −2.71, −0.14, *p* = 0.032) but not among more-fit individuals (β = −0.17, 95% CI = −0.59, 0.25, *p* = 0.396). The same pattern was evident using robust regression (not shown). As detailed in Table 2, the sleep × fitness effects were not evident for the remaining sleep metrics (TST and WSTP) (FDR-corrected *p*s ≥ 0.108) or amygdala volume (*p*s ≥ 0.227). 

### 3.3. Greater Sleep Consolidation Was Associated with Larger Hippocampal Volume

Across the fitness groups, a greater sleep consolidation (WSTP) was associated with a larger hippocampus (β = 0.52, 95% CI = 0.16, 0.89, FDR-corrected *p* = 0.037) (Figure 3b). Simple bivariate sleep–brain relations were not evident for sleep duration (TST) (FDR-corrected *p*s ≥ 0.423) or amygdala volume (*p*s ≥ 0.064) (Table 2). 

### 3.4. Cardiorespiratory Fitness Moderated Relations between Sleep Duration and Global Cognition

**Figure 4:**
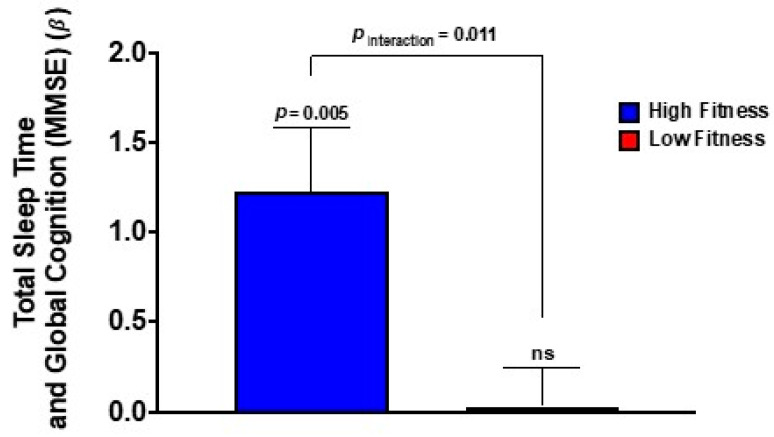
**Relations between sleep, fitness, and global cognition in older adults.** Cardiorespiratory fitness (HRR_M_) moderates the positive association between sleep duration and global cognition. Bars depict standardized regression coefficients (β) for the sleep × fitness interaction (controlling for mean-centered age) for more-fit (*blue*) and less-fit (*red*) individuals. Among more-fit individuals (*blue*), longer sleep duration is associated with better global cognition. This association was not significant for less-fit individuals (*red*). Error bars depict standard error.

Regression analyses indicated that variations in cardiorespiratory fitness (HRR_M_) also moderated relations between sleep duration (TST) and global cognition (MMSE) (β = −1.07, 95% CI = −1.70, −0.43, FDR-corrected *p* = 0.011). As shown in Figure 4, stratified analyses demonstrated that a longer TST was associated with enhanced cognition among more-fit individuals (β = 1.23, 95% CI = 0.45, 2.01, *p* = 0.005) but not less-fit individuals (β = 0.03, 95% CI = −0.44, 0.50, *p* = 0.891). As shown in Table 2, the sleep × fitness interaction effects were not evident for the remaining sleep metrics (WBL and WSTP) (FDR-corrected *p*s ≥ 0.191). 

### 3.5. Longer Wake Bout Length Was Associated with Lower Global Cognition

Across the fitness groups, a longer wake bout length (WBL) was associated with lower MMSE scores (β = −0.40, 95% CI = −0.73, −0.07, FDR-corrected *p* = 0.042). Simple bivariate sleep–cognition associations were not evident for sleep consolidation (WSTP) (FDR-corrected *p*s ≥ 0.107) (Table 2).

## 4. Discussion

Older adults with sleep problems are at increased risk of cognitive decline and dementia. Chronic aerobic exercise training, known to improve and maintain cardiorespiratory fitness, represents a feasible intervention to improve both sleep and brain health [54]. In this study of physically active, cognitively normal adults aged 55 and older, we addressed the possible moderating effects of cardiorespiratory fitness on the relationships between sleep and regional brain volume and sleep and global cognitive performance in older adults. Our findings revealed that a longer WBL was associated with smaller hippocampal volume among those with lower (slower HRR_M_) but not higher fitness (faster HRR_M_). Our results also demonstrated that higher fitness amplifies the benefits of longer sleep duration on global cognitive performance, suggesting that higher levels of cardiorespiratory fitness may safeguard the hippocampus and cognition from sleep-related changes in older persons, among whom poor sleep is a common complaint. Further, regardless of fitness, individuals with more consolidated sleep (higher WSTP) exhibited larger hippocampal volume, and individuals with a longer wake bout length (longer WBL) had poorer global cognitive performance, providing additional evidence for the neurocognitive consequences of poor sleep later in life. 

Our findings linking sleep disturbance with smaller hippocampal volume are broadly consistent with those from the Rush Memory and Aging Project study, which found that greater sleep fragmentation—measured by actigraphy—was associated with smaller total cortical gray matter volume and smaller regional gray matter volume in the bilateral orbitofrontal cortices and inferior frontal gyri pars orbitalis [6]. A second longitudinal investigation of data in the Baltimore Longitudinal Study of Aging found that individuals with <7 h of self-reported sleep duration had higher rates of subsequent thinning in the left superior temporal, inferior and middle frontal cortices, and right superior frontal cortex compared to those reporting 7 h of sleep, whereas those with >7 h had higher rates of thinning in the left superior and middle frontal cortices [7], suggesting that both shorter and longer sleep durations may have negative neurocognitive consequences in older populations. Our smaller sample size and limited range of sleep duration did not permit us to adequately examine its potential nonlinear (inverted U-shaped) associations with hippocampal volume or global cognition. Notwithstanding, our findings are consistent with the literature that suggests poor sleep quality may play an integral role in age-related neurocognitive degeneration [3,5,23]. The results from the current study revealed sleep-related differences in hippocampal volume based on hypothesis-driven analyses that were selected *a priori* based on the well-established neurotrophic effects of exercise on the hippocampus. We also demonstrated that these effects were specific to the hippocampus and did not generalize to the amygdala, another medial temporal lobe structure; we did not interrogate the cortical gray matter. 

Our results regarding the fitness-related preservation of hippocampal volume in the context of sleep disturbance are compelling as several prior studies have identified a salubrious impact of exercise and/or improvements in fitness on brain volume and structural integrity in later life [13,25,26,28,29,30]. One such study found that higher aerobic fitness was associated with larger hippocampal volume [24], whereas another study found that higher fitness was linked to reduced age-related brain atrophy in the frontal, parietal, and temporal cortices [27]; and a 12-week exercise intervention in a sample comprised of both older adults with normal cognition (*n* = 16) and individuals with mild cognitive impairment (*n* = 14) demonstrated that increased cardiorespiratory fitness was associated with greater cortical thickness after exercise training in the bilateral insula, precuneus, posterior cingulate, precentral gyri, and inferior and superior frontal cortices [55]. However, the answer to the question of how greater fitness would protect brain tissue volume from the deleterious effects of poor sleep remains unclear. Our study also does not address the possibility that better sleep leads to an opportunity to achieve higher fitness [56]. To our knowledge, however, only two other studies have examined the interactive effects of sleep and fitness on the markers of neurodegeneration. Using data from 74 older adults in the Wisconsin Registry of Alzheimer’s Prevention, an interaction was reported between self-reported sleep quality—measured by the Medical Outcomes Study Sleep Scale—and aerobic fitness on CSF levels of total tau (t-tau), phosphorylated tau (p-tau), and the p-tau/β-amyloid_42_ ratio, in which poor sleep was associated with greater neurodegeneration among older individuals with lower but not higher cardiorespiratory fitness [21]. The other study, which was conducted using data from the Wisconsin Sleep Cohort (and currently only published in abstract form), demonstrated an adverse relationship between sleep apnea severity and total gray matter volume among less-fit (but not more-fit) cognitively normal older individuals [8]. Although our study was different in both measurement and design, our results demonstrating the modifying effects of HRR_M_ on hippocampal volume in disturbed sleepers are congruent with the findings from this report. 

### 4.1. Potential Mechanisms

The neurophysiological mechanisms related to the association between sleep and brain volume may also help account for the neuroprotective effects of higher cardiorespiratory fitness in the face of poor sleep including growth factors, pro-inflammatory cytokines, and clearance of metabolic waste. For example, insomnia and abnormal sleep duration have been associated with lower circulating levels of BDNF [57], which protects against age-related brain atrophy [58], whereas both endurance exercise training and higher cardiorespiratory fitness have been shown to increase levels of BDNF [59]. Similarly, a shorter sleep duration and sleep fragmentation have been tied to pro-inflammatory cytokines, such as C-reactive protein (CRP) and interleukin-6 (IL-6) [60], which have been inversely associated with hippocampal volume [61], whereas aerobic exercise training and fitness improvements have been shown to reduce levels of CRP and IL-6 [62]. Additionally, the glymphatic system, which is more active during deep non-rapid eye movement (NREM) sleep, facilitates the clearance of metabolic waste [63] including both amyloid-β [64] and tau protein [65]. It is also possible that sleep disturbances (i.e., longer WBL) result in lower levels of slow-wave activity during NREM sleep and may thereby inhibit glymphatic function, thus hindering the clearance of neurotoxic substances (e.g., β-amyloid, tau, α-synuclein) and ultimately promoting neurodegeneration. Although more research is warranted, recent evidence indicates that aerobic exercise training improves sleep [66] and may even enhance glymphatic system function [67], highlighting the pleiotropic nature of exercise and yet another pathway by which exercise training may improve cognition and slow the progression of neurodegenerative disease. Conversely, it is also plausible that good sleep increases next-day physical activity [56] and/or protects against the negative effects of low fitness; both of which underscore the need for future studies in this area. 

### 4.2. Future Challenges

The strengths of this study include the sample of physically active, cognitively normal older adults, who completed wrist actigraphy (~8 days), a single bout of moderate-intensity exercise from which HRR_M_ was quantified, and an MRI scan, which allowed us to evaluate the influence of objectively measured sleep and HRR_M_ on regional brain volume. Further, by combining data from repeated MRI scans, we improved image reconstruction and the identification of tissue-specific and regional boundaries. Despite these strengths, the present study is not without limitations. Although we used wrist actigraphy to measure sleep, we had no objective indices of sleep stages or sleep-disordered breathing, which limited the scope of our sleep analyses. Similarly, although we used HRR_M_ as a proxy for aerobic fitness, a more direct measure of fitness (e.g., maximal or sub-maximal graded exercise tests) should be used in future studies. Nevertheless, a faster HRR_M_ (higher fitness) was associated with performing exercise at a lower percentage of maximal capacity, indicating that the overall metabolic and cardiorespiratory strain associated with ‘Hard’ exercise was less in this group, and could thus be performed at a relatively lower percentage of maximal capacity (exercise intensity as a percentage of HR_Reserve_). Such findings, in association with the established literature on HR return towards baseline after exercise [42,43,44,45,46,47], suggest that HRR_M_ provides a reasonable proxy for cardiorespiratory fitness. Due to the rigor of our eligibility criteria, future studies using larger, more diverse samples—including separate studies focused on older adults with cognitive impairments—would enhance the generalizability of the results. Because of our cross-sectional study design and the fact that we manipulated neither fitness nor sleep, we cannot determine the causal directionality of or the driving factors underlying these relationships. Thus, another crucial direction for future research would be to manipulate sleep (e.g., via acute, partial, and full deprivation and sleep-enhancement or extension sessions) and physical (in)activity (e.g., via exercise sessions or periods of sedentary behavior/detraining) to clarify their mechanistic importance in the context of brain health among older adults. Finally, future larger-scale studies should (1) evaluate the specific sub-regions of the hippocampus using similar experimental procedures to determine whether distinct hippocampal regions (anterior/head, body, posterior/tail) exhibit a differential response to poor sleep; and (2) seek to determine whether the potential protective effects of fitness on sleep-related health outcomes differ by biological sex.

## 5. Conclusions

We found that cardiorespiratory fitness (HRR_M_) moderated the associations of sleep with hippocampal volume and global cognition. Specifically, a longer wake bout length (longer WBL) was linked with smaller hippocampal volume among individuals with lower but not higher fitness, and a longer sleep duration was associated with better global cognitive performance among individuals with higher but not lower fitness. Additionally, regardless of fitness level, our results indicated that more consolidated sleep (WTSP) was linked to larger hippocampal volume and that more disrupted sleep (longer WBL) was associated with poorer global cognition. Overall, these findings suggest that sleep may play an important role in maintaining the structural integrity of brain regions critical to learning and memory and that higher levels of fitness may protect against the impacts of disrupted sleep in old age, setting the stage for scalable interventions. 

## Figures and Tables

**Table 1:** Participant characteristics.

Characteristic	Mean ± SD
Age, years	65.8 ± 7.3
Female	22 (73.3)
Education > 12 years	28 (93.3)
White	23 (76.7)
Body Mass Index, kg/m^2^	25.2 ± 4.0
7-Day PA, kcal/kg/day	223.3 ± 28.1
Actigraphy, days	7.9 ± 3.4
Total Sleep Time, min	403.6 ± 71.9
Wake Bout Length, min	5.7 ± 2.8
Wake-to-Sleep Transition Probability	0.2 ± 0.1
Borg’s Rating of Perceived Exertion	14.6 ± 1.2
Baseline Heart Rate, bpm	76.9 ± 10.1
Post-Exercise Heart Rate, bpm	92.8 ± 13.5
Exercise Intensity as a Percentage of HR_Reserve_, %	73.7 ± 20.7
Hippocampal Volume, mm^3^/ICV	0.0052 ± 0.0006
Amygdala Volume, mm^3^/ICV	0.0020 ± 0.0002
Mini-Mental State Exam	29.1 ± 1.2

*Note*. Female, education, and white are expressed as *n* (%). The Borg’s Rating of Perceived Exertion and all heart rate metrics reflect measurements during the exercise condition. *Abbreviations.* PA—physical activity, HR_Reserve_—heart rate reserve (calculated as max HR—resting HR), SD—standard deviation, kg—kilograms, m—meters, min—minutes, kcal—kilocalories, bpm—beats per minute, ICV—intracranial volume.

**Table 2:** Sleep × fitness (HRR_M_) interaction for regional brain volume and global cognition.

	Neurocognitive Outcomes		
Parameter	Hippocampal Volume	Amygdala Volume	Global Cognition (MMSE)
	β (95% CI)	β (95% CI)	β (95% CI)
WBL	---	−0.09 (−0.50, 0.32)	−0.40 (−0.73, −0.07) *
WBL × Fitness	−1.36 (−2.45, −0.28) *	−0.74 (−1.98, 0.49)	0.51 (−0.46, 1.48)
			
WSTP	0.52 (0.16, 0.89) *	0.31 (−0.10, 0.72)	0.33 (−0.06, 0.73)
WSTP × Fitness	0.60 (−0.12, 1.33)	0.23 (−0.61, 1.08)	−0.44 (−1.17, 0.29)
			
TST	0.11 (−0.33, 0.55)	0.40 (−0.03, 0.82)	*---*
TST × Fitness	−0.15 (−1.07, 0.77)	−0.31 (−1.19, 0.56)	−1.07 (−1.70, −0.43) *

*Note.* Significant results are in bold and indicated by the following: * *p* < 0.05. Main effects were not evaluated for models demonstrating a significant interaction, denoted by the dashed line (---). *Abbreviations.* TST—total sleep time, WBL—average wake bout length, WSTP—wake-to-sleep transition probability, HRR_M_—modified heart rate recovery, MMSE—Mini-Mental State Exam, β—standardized beta coefficient, CI—confidence interval.

## Data Availability

The data reported in this paper are available upon request from any qualified investigator for reanalysis and replication of results.
